# Phosphonated Polyetheramine-Coated Superparamagnetic Iron Oxide Nanoparticles: Study on the Harsh Scale Inhibition Performance of Calcium Carbonate and Barium Sulfate

**DOI:** 10.1021/acsomega.4c07018

**Published:** 2024-09-29

**Authors:** Ali H. Karaly, Malcolm A. Kelland, Mohamed F. Mady

**Affiliations:** † Department of Chemistry, Bioscience and Environmental Engineering, Faculty of Science and Technology, University of Stavanger, N-4036 Stavanger, Norway; ‡ Department of Chemistry and Earth Sciences, College of Arts and Sciences, Qatar University, P.O. Box 2713, Doha, Qatar

## Abstract

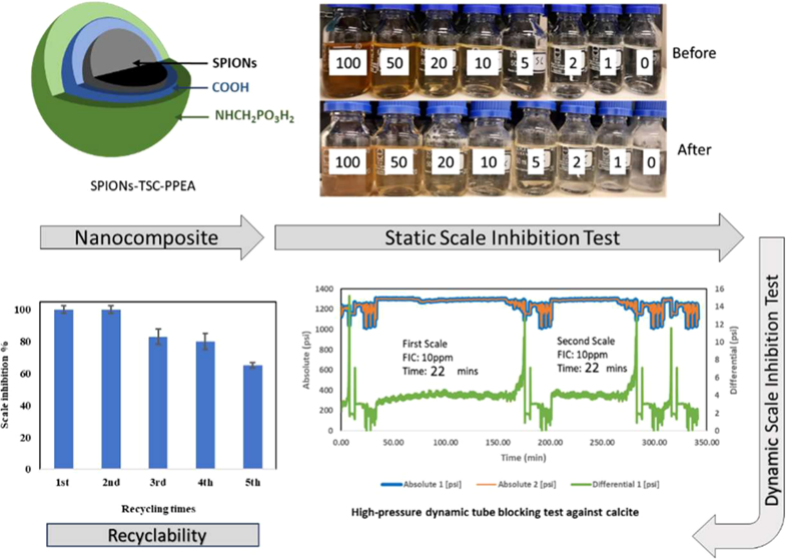

Harsh scale buildup, such as calcium carbonate (calcite) and barium sulfate (barite), poses significant challenges in the oil and gas industry. While various scale inhibitors (SIs) are employed to mitigate this issue, there is a need for greener, more efficient, compatible, and affordable alternatives. Calcium compatibility often complicates the use of SIs, potentially leading to formation damage. Our group has recently synthesized a recyclable nanocomposite scale inhibitor made of superparamagnetic nanoparticles coated with a trisodium citrate linker to phosphonated poly(ether amine) (SPION-TSC-PPEA) and tested it against calcium sulfate (gypsum), a simple form of scale buildup. This study evaluates the recyclable nanocomposite scale inhibitor’s efficiency in mitigating calcite and Barite scales through static jar tests and high-pressure dynamic tube-blocking tests at 80 bar and 100 °C. The nanocomposite demonstrated high calcium ion compatibility, excellent inhibition efficiency against calcite, moderate efficiency against Barite, and maintained efficacy over five recycling cycles.

## 1 Introduction

In the offshore upstream oil industry, the buildup of inorganic salt from field water composition can block pore throats in the well itself or the near-wellbore region.^1,2^ This process is called scaling, and once a scale layer is formed, it grows to cause formation damage and reduce the well productivity unless treated.^3^ Scale formation occurs due to salt supersaturation; when the activity of cations and anions of salt exceeds their saturation limit, the crystallization process starts to occur and scale deposition starts to form. The scaling process is complex, as it involves several crystallization mechanisms. The kinetics of the reaction is a factor in the degree of scaling.^4,5^ There are several common types of scales in the oil and gas industry, such as calcium carbonate (CaCO_3_, calcite, and aragonite), sulfates of group II elements such as strontium (SrSO_4_, celestite), calcium (CaSO_4_·2H_2_O, gypsum), and barium (BaSO_4_, barite).^4^

Calcite forms when water containing high concentrations of calcium ions mixes with water rich in carbonate ions, a common occurrence in the production and injection processes. The decrease in the pressure and temperature during oil extraction can also lead to the precipitation of calcite. At higher pH levels, there is a greater concentration of carbonate ions (CO_3_^2–^) due to the dissociation of bicarbonate (HCO_3_^–^). This increase in CO_3_^2–^ enhances the likelihood of calcium carbonate precipitation according to the following chemical equation:
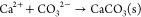
1

This reaction occurs when calcium-rich water (e.g., formation water) mixes with water that contains high concentrations of carbonate ions, often due to the dissolution of carbon dioxide (CO_2_) in water. Barite, on the other hand, precipitates when barium ions in the formation water interact with sulfate ions from injected seawater or formation water itself according to the following equation:
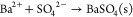
2

Its precipitation is less directly influenced by pH compared to calcite because sulfate (SO_4_^2–^) ions are less affected by pH changes within the typical range encountered in oilfield waters. The conditions in the subsurface environment, such as high pressure and temperature, exacerbate the deposition of these scales.^4^

In the oil and gas industry, forming calcite (calcium carbonate) and barite (barium sulfate) scales presents significant operational challenges and economic drawbacks. Both scale types can severely reduce flow efficiency by constricting pipelines and production tubing, leading to increased pressure drops and energy consumption. They cause equipment damage due to their hard and abrasive nature, necessitating frequent maintenance and costly replacements. Scale buildup requires regular management, which results in increased maintenance costs, operational downtime, and production interruptions. Barite scale, in particular, poses severe blockage risks and is resistant to many conventional chemical treatments, complicating its management. The processes for scale treatment involve health and safety risks and environmental concerns due to the disposal of scale-laden fluids and chemicals. Consequently, effective scale management strategies are essential to mitigate these issues and maintain smooth, cost-effective operations.^6^

Several techniques inhibit the scale formation and development of scale crystals. Mechanical treatment is one of the conventional methods to remove the inorganic scale deposits in the wellbore tubular.^5,7^ Chemical treatment can also remove the formed scale through several techniques, such as chelating agents or acid stimulation, and it is used in the upstream oil and gas industry mainly for carbonate scale removal. However, mechanical and chemical dissolution methods have disadvantages such as high costs for pipeline corrosion and environmental issues. Scale inhibitors are chemical additives used in the oil and gas industry to prevent the formation of mineral scales such as calcium carbonate and barium sulfate on production equipment, pipelines, and reservoir formations. These inhibitors work by interfering with the crystal growth process of scale-forming minerals, keeping them in solution, or altering their structure to prevent deposition. Scale inhibitors offer significant advantages compared with other methods of scale management, such as mechanical removal or periodic acid cleaning. They provide continuous protection against scale buildup, reducing the need for frequent maintenance and associated downtime. This leads to enhanced production efficiency and reduced cost savings. Additionally, scale inhibitors can be precisely tailored to specific operational conditions, making them more versatile and practical across various environments. Their preventive approach helps to maintain optimal flow rates and extend the lifespan of equipment, thereby ensuring smoother and more reliable oil and gas production operations.^8−10^

The main functional groups in the SIs are mainly carboxylate, phosphonate, and sulfonate functional groups.^11^ The latter two are used for downhole and topside oilfield scale control. Organophosphorus compound-based SIs, such as aminomethylenephosphonate groups, are more explicitly used for various scale types in the upstream oil and gas industry. The latter group is believed to enhance the metal-binding ability of the molecule via amine and phosphonate interaction, improving the SI’s chelating ability.^12−16^ In addition, their concentrations in the formation water can be calculated by analytical and spectroscopic techniques.

**Figure 1:**
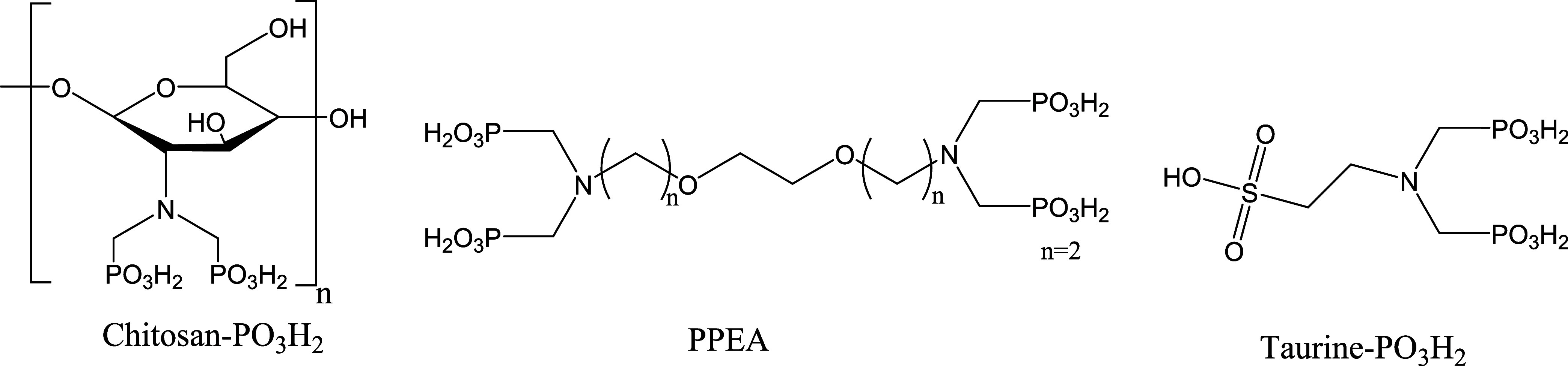
Environmentally accepted SIs developed by our group.

However, most of the typical industrial products of phosphonate SIs have poor biodegradability as well as poor compatibility with high concentrations of calcium ions, which leads to precipitation of the SI as a complex, such as diethylenetriaminepentakis (methylenephosphonic acid) (DTPMP) and amino tris(methylenephosphonic acid) (ATMP).^17^ Accordingly, and to the obsessive need for greener and compatible SIs, many commercial SIs were prohibited in the U.K. and Scandinavia to match the offshore updated regulation determined by the Oslo and Paris Commission (OSPARCOM).^18,19^ During the current decade, our Green and Sustainable Chemistry research group managed to design new green SIs for oilfield applications such as chitosan-PO_3_H_2_, phosphonated polyertheramine (PPEA), and taurine- PO_3_H_2_, as shown in Figure 1. One of the recent studies showed that PPEA are efficient green SIs against calcite and Barite scales with good calcium compatibility and thermal stability.^20^

Currently, environmentally friendly SIs suffer from certain drawbacks such as low efficiency, high cost, and several incompatibilities with the production system, as it is not easy to tick all of the advantages boxes. So, there is a great need to have a new technique to avoid chemical pollution from oilfields to the natural aqueous environment.

Nanoscience has attracted attention due to its various potential in different applications.^21^ In the upstream oil and gas application, nanoscience also proved efficient.^21^ Our research group recently developed a superparamagnetic iron oxide nanoparticles (SPIONs) composite covered with polymeric SI.^22^ The combination of SPIONs and SIs can open new prospects of having recyclable SIs on a commercial level that will reduce the overall cost and significantly lessen the environmental impact of the discharged solution. This composite was proved to be efficient and recyclable against the Gypsum scale, which is a mild scale relative to heidrun calcite and barite.^22^ This study aims to reach a zero environmental impact from the SIs by attaching the used chemicals to SPIONs to synthesize an efficient, compatible, recoverable, recyclable SI composite that is efficient in harsh environments such as the Heidrun oilfield, Norwegian Sea, Norway.

In this study, the prepared green nanocomposite from our recent study was applied to harsh environments for the first time with zero environmental impact. The composite was tested against calcite and barite using a dynamic scale rig and a static inhibition test. The composite’s recyclability is assessed up to five times. In addition, the composite’s calcium compatibility will be assessed through visual observation.

## 2 Experimental Section

### 2.1 Materials and Characterization

The chemicals and solvents mentioned below were utilized without further modification and used as they were, unless otherwise mentioned. They were obtained from VWR Chemicals, ACROS Organics, Tokyo Chemical Industry Co., Ltd., and Sigma-Aldrich (Merck). ATMP commercial SI was purchased from Italmatch Chemicals S.p.A. Italy.

In addition, these devices have been used: a phenomenal pH 1100 L VWR, a Z671797 IKA C-MAG HS hot plate stirrer, a pH/mV/°C meter, a bench, a Bransonic ultrasonic cleaner, an automated Thermo Fisher Scientific, and Milli-Q water obtained from ELGA PURELAB Option-R 7.

### 2.2 Synthesis and Characterization of Polymers and Nanoparticles

The synthesis of the SPION-TSC-PPEA nanocomposite was carried out as detailed in our previous research.^23^ The magnetic core of the nanocomposite, superparamagnetic iron oxide nanoparticles (SPIONs), was prepared via chemical coprecipitation. Subsequently, a trisodium citrate linker layer was applied by using sonication, followed by a washing step. The PPEA SI polymer was then added to the system, also using sonication, and subsequently washed. Comprehensive characterization of the nanocomposite’s morphology, elemental composition, chemical functionalization, crystal structure, and magnetic properties has been documented in our prior study.^23^

### 2.3 Testing of SI Performance

#### 2.3.1 Static Jar Test

The static jar test is one of the methods used to assess the performance of the synthesized nanoparticles in inhibiting oilfield scale crystal growth. The static inhibition test has been standardized according to NACE protocol TM0374-2007.^24^ It demands the presence of three main elements: anion solution, cation solution, and tested chemical in decreasing concentrations.^25^ The test has been performed on the calcite scale as prepared in Table 1 according to the Heidrun oilfield, Norwegian Sea, Norway. The composition of Heidrun brines (1:1 mixture between formation water and seawater) is given in Table 2.

**Table 1:** Composition of Brines for Calcite Scaling by the NACE Standard TM0374-2007 Protocol^24^

ion	conc (ppm)	component	brine 1 (g/L)^a^	brine 2 (g/L)^b^
Na^+^	25,964	NaCl	33.000	33.000
Ca^2+^	3314	CaCl_2_·2H_2_O	12.150	0
Mg^2+^	440	MgCl_2_·6H_2_O	3.680	0
HCO_3_^–^	5346	NaHCO_3_	0	7.360

a pH of brine 1 (B1) is 5.5.

b pH of brine 2 (B2) is 7.1.

**Table 2:** Composition for Calcite Scaling Based on Heidrun Oilfield Produced Water

ion	conc (ppm)	component	brine 1 (g/L)^a^	brine 2 (g/L)^b^
Na^+^	39,020	NaCl	49.590	49.590
Ca^2+^	2040	CaCl_2_·2H_2_O	7.480	
Mg^2+^	530	MgCl_2_·6H_2_O	4.430	
K^+^	1090	KCl	2.078	
Ba^2+^	570	BaCl_2_·2H_2_O	1.014	
Sr^2+^	290	SrCl_2_·6H_2_O	0.882	
HCO_3_^–^	1000	NaHCO_3_	0	2.760

a pH of B1 is 5.5.

b pH of B2 is 7.1.

In 50 mL Schott Duran glass bottles, the calcite scale produced a 1:1 solution of 20 mL of B1 and B2 with a total volume of 40 mL. Different concentrations of the SI were dosed to the brine solution to test its scale inhibition performance by diluting a 1000 ppm of SI stock solution with pH 4.5 mimicking the oil reservoir, as shown in Table 3. Volumes of SI were added using automated Thermo Fisher Scientific, 100 μL, 1-, and 10 mL pipettes.

**Table 3:** Different Concentrations of Static Inhibition Performance of SI

SI concentration (ppm)	B1 (mL)	B2 (mL)	1000 ppm stock SI (mL)
100	20	16	4
50	20	18	2
20	20	19.2	0.8
10	20	19.6	0.4
5	20	19.8	0.2
2	20	19.92	0.08
1	20	19.96	0.04
0	20	20	0

Bottles were mixed, closed, and heated to scale over 5 h in the oven at 80 °C for all samples (except positive control blank).^20,26^ Then, all samples were tested to determine the concentration of Ca^2+^ in the solution by EDTA titration and the presence of Ca^2+^ would reflect scale inhibition, hence, SI efficacy. Titrations have been performed in triplicates, and the standard deviation was below 5%. The titration protocol has been conducted as given by ASTM D-511.^26^ The titration setup consisted of a lab disk stirrer (VWR, Germany) and a 25:0.05 mL glass buret (Hirschmann, Germany). Typically, 1 mL aliquot of each sample was withdrawn and diluted with 50 mL of DI water in an Erlenmeyer flask. The pH was adjusted by adding 100 μL of 50% NaOH and 200 μL of the indicator. EDTA concentration was set to 0.1 M, and the color changed from yellowish green to orange upon the endpoint. The volume of the titrated EDTA was recorded and used to calculate the Ca^2+^ concentration through eq 3
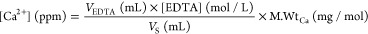
3where *V*_EDTA_ is the volume of consumed EDTA, [EDTA] is set to 0.1, representing the EDTA concentration, *V*_S_ is set to 50, representing the sample volume, and M.Wt_Ca_ is set to 40,100, representing the molecular mass of calcium.

The scale inhibition efficiency can be calculated, giving the Ca^2+^ concentration as eq 4
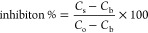
4where *C*_s_ refers to the calcium concentration in each sample after treatment, *C*_b_ is the concentration in the blank after treatment, and *C*_o_ is the concentration in the original blank solution before treatment.

#### 2.3.2 High-Pressure Dynamic Tube-Blocking Test

The high-pressure dynamic tube-blocking test has proven to be an efficient lab-scale method for mainly testing the scale inhibition effects of SIs for oil and gas applications. This method mimics the condition of inorganic scaling in the real field regarding pressure and temperature.^20,27−29^ The dynamic tube-blocking scale rig is a device that mimics the scale formation process inside the pipelines in terms of pressure and temperature, and the used one is manufactured via Scaled Solutions Ltd.

Scaled Solutions Ltd. (U.K.) manufactured the dynamic tube-blocking scale rig. The operation mimics the scale formation process inside the pipelines regarding the pressure and temperature and measures the SIs efficiency. The differential pressure values across the coil indicated the scale formation rate. So, it is attached to a computer that compiles the differential pressure data to determine the fail inhibitory concentration (FIC) and minimum inhibitory concentration (MIC) at which the scaling occurs for different SIs. Scale growth in the coil leads to an increase in the differential pressure across the coil, triggering the stop of the reaction at a differential pressure set over 14 psi. The coil is designed to withstand temperature and pressure of up to 200 C and 300 bar.

**Figure 2:**
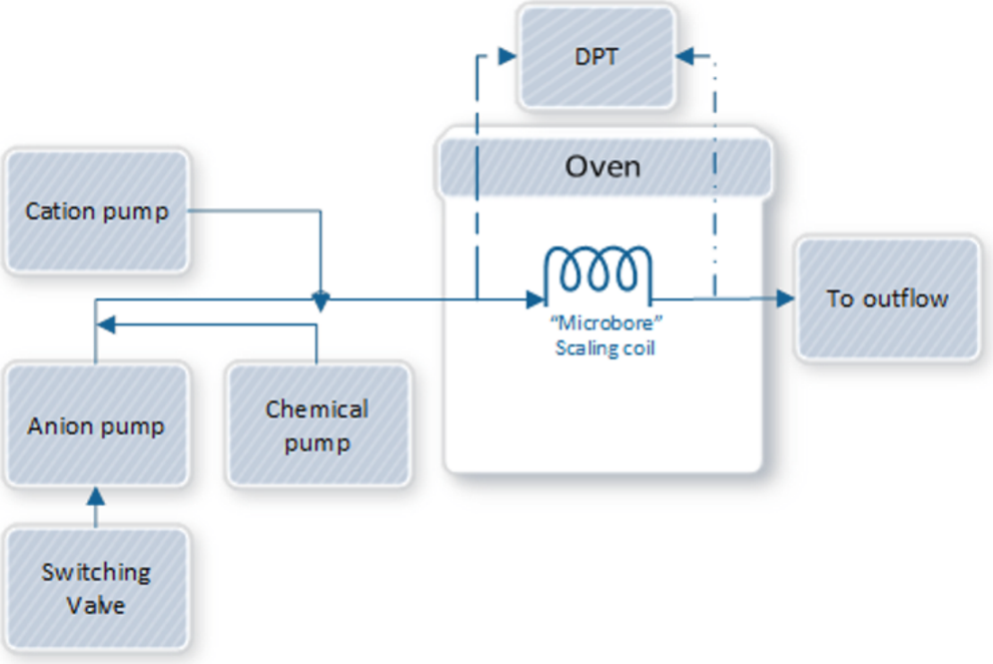
Schematic representation of the dynamic tube-blocking equipment. Reproduced with permission from ref (20). Copyright^©^ 2020 American Chemical Society.

As shown in Figure 2, the rig consists of three pumps. Pump 1 injects brine 1, pump 2 is multivalved and used to inject brine 2, an EDTA scale-removing solution (a high-pH solution of tetrasodium ethylenediaminetetraacetate (Na_4_EDTA 5 wt %, pH = 11–13) in Milli-Q water) or DI water used for cleaning, depending on the chosen valve, and pump 3 injects SI. The water used in the mentioned solutions is DI water, and the solutions were degassed before injection and injected into the coil at 100 °C and 80 bar.

The automated operation protocol was adjusted to four main stages as follows:

(1) Blank test without SI
(2) A series of decreasing concentrations of SI, lasting for 1 h each.
(3) A repeated test of the MIC and FIC.
(4) A repeated blank test.

The starting concentration of the SI is set to 100 ppm and descends to 50, 20, 10, 5, 2, and 1 ppm, unless the FIC is reached. During FIC, the scale blocks the coil. So, a cleaning sequence is set to ensure the coil is usable. First, the EDTA solution will pass in the coil for 10 min as a scale removal. Then, the DI water is passed for 10 min to neutralize the coil. An additional step is added with SPION composites as SI, which is 10% acetic acid washing. All of the results are compiled into Excel files and processed as schematic graphs of the scaling time in minutes against the differential pressure (psi) inside the stainless-steel coil.

### 2.4 Calcium Compatibility Test

The scale inhibition efficiency of an SI can be hindered by divalent cations such as Mg^2+^ and Ca^2+^ present in the formation water and the barium sulfate saturation ratio.^30^ A compatibility test is performed to check the SI compatibility with Ca^2+^ presence and to avoid formation damage of the SI. Typically, several solutions with different SI concentrations (100, 1000, 10,000, and 50,000 ppm) are mixed with a high concentration of Ca^2+^ (10,000 ppm) in the presence of 3 wt % of NaCl in 20 mL of DI water inside 50 mL Duran glass bottles. The pH of all solutions was adjusted to the range of 4.0–4.5. The bottles were shaken to reach a homogeneous solution and placed in the oven at 80 °C for 24 h, with visual inspection at 30 min, 1, 4, and 24 h. The compatibility of the composite will be assessed using visual observation of the solution mixture after the located time interval.

## 3 Results and Discussion

### 3.1 Static Inhibition test

**Figure 3:**
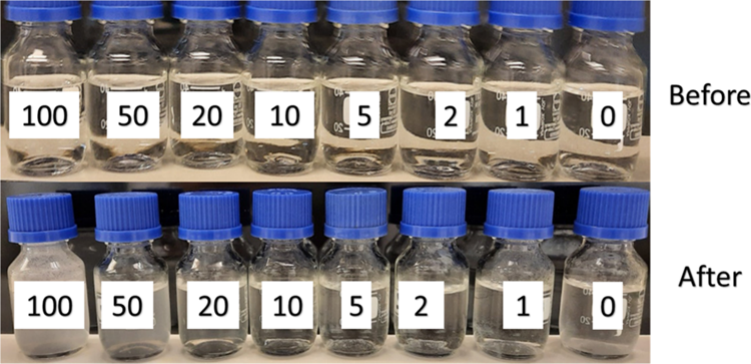
Static inhibition test of different concentrations of ATMP before and after 5 h at 80 °C.

**Figure 4:**
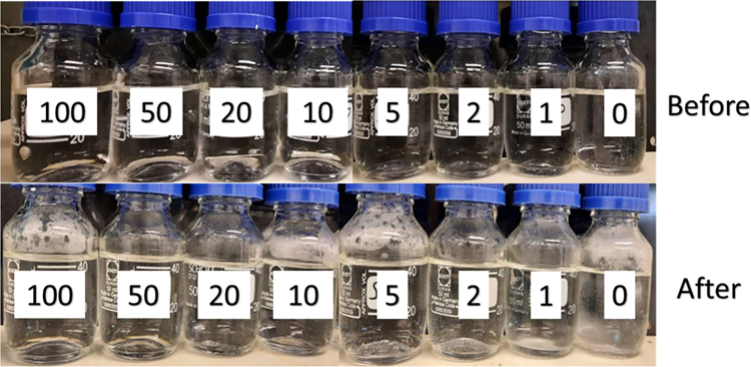
Static inhibition test of different concentrations of PPEA before and after 5 h at 80 °C.

**Figure 5:**
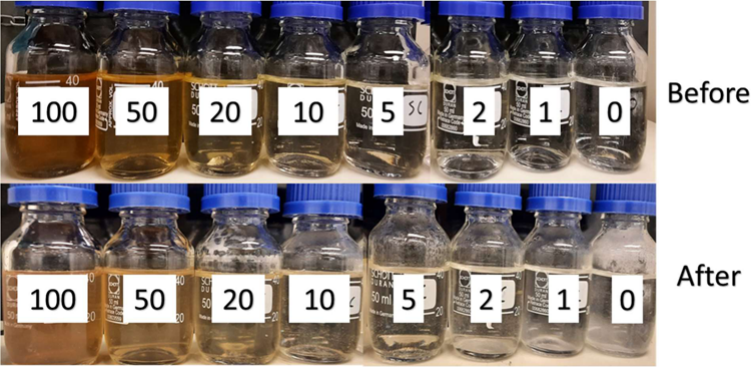
Static inhibition test of different concentrations of SPION-TSC-PPEA before and after 5 h in 80 °C.

Static inhibition tests of the PPEA polymer and synthesized SPION compound were performed against calcite compared with the ATMP commercially available SI, as shown in Figures 3–5, respectively. The PPEA polymer had a better performance than the ATMP commercial polymer. The PPEA gave total inhibition in concentrations 100 and 50 ppm, and then, it was still high inhibition performance at 20 ppm concentration by 91%. It decreased to acceptable levels at 10 and 5 ppm concentrations by inhibition performance of 85 and 81%, respectively. At low concentrations of 2 and 1 ppm, the inhibition performance fell dramatically to 50 and 18%, respectively. The ATMP is known to have compatibility problems with Ca^2+^, which can illustrate its increase in efficiency at lower concentrations.^31−34^ The inhibition efficiency of ATMP was low in high concentration, as shown in Table 4, due to Ca^2+^ compatibility problems apparent by the white turbidity, as shown in Figure 3. Ca^2+^ compatibility problems decrease with decreasing the concentration of the ATMP. So, the turbidity of the solution after 5 h in the oven decreases with decreasing concentrations as well, and scale inhibition efficiency increases from 71% at 100 ppm to 100% at 5 ppm. ATMP inhibition efficiency decreases again with lower concentrations to 91 and 88% at 2 and 1 ppm, respectively.

**Table 4:** Calcite scale inhibition performance of SPION-TSC-PPEA, and ATMP, according to NACE protocol TM0374-2007 to Heidrun oilfield, Norway

	PPEA		SPION-TSC-PPEA		ATMP	
conc. (ppm)	average inhibition, %	SD^a^	average inhibition, %	SD^a^	average inhibition, %	SD^a^
100	97	4	97	4	71	3
50	97	4	95	2	84	4
20	91	2	92	0	88	3
10	85	0	92	0	91	3
5	81	4	73	4	100	4
2	50	4	35	4	91	3
1	18	4	13	4	88	3

a SD: Standard Deviation.

The SPION composite gave a behavior similar to that of the PPEA in calcite scale inhibition efficiency. The composite even performed better than the PPEA polymer at 10 ppm. The higher efficiency of SPION composite over PPEA polymer can be illustrated by the effect of nanoparticle huge surface area, which can increase the efficiency of the SI polymer.^35^

The mechanisms of scale inhibition against barite and calcite can differ through several stages of scale formation. Phosphonates exhibit a threshold effect, maintaining ions like calcium and barium in solution even when their concentrations exceed solubility limits, thus preventing precipitation.^36^ Moreover, phosphonates are known to behave as sorption isotherms.^37^ This means that there is a linear relationship between the concentration of the phosphonates and their inhibition efficiency by adsorbing onto the scale potential nuclei, reducing the availability of active sites needed for crystal growth. One of our previous density functional theory studies showed that more phosphonates on the chemical backbone of the SI can lead to a stronger affinity toward calcite and barite.^38^ In this context, the four phosphonate groups in the PPEA exhibit SI activity superior to that of the three phosphonates in the ATMP structure backbone.

From this perspective, we can predict that SPION-TSC-PPEA have the same inhibition mechanism due to PPEA coating. However, it should be mentioned that the nanoparticles can act as scale nucleation points themselves, as shown by the SEM images of gypsum crystals with nanoparticles deposited onto them in our previous study.^23^ This is why the sonication is a very crucial part of the washing steps to make sure not to have any deposited nanocrystals on the surface of SPION-TSC-PPEA upon recycling.

### 3.2 Recyclability of the Composite

**Figure 6:**
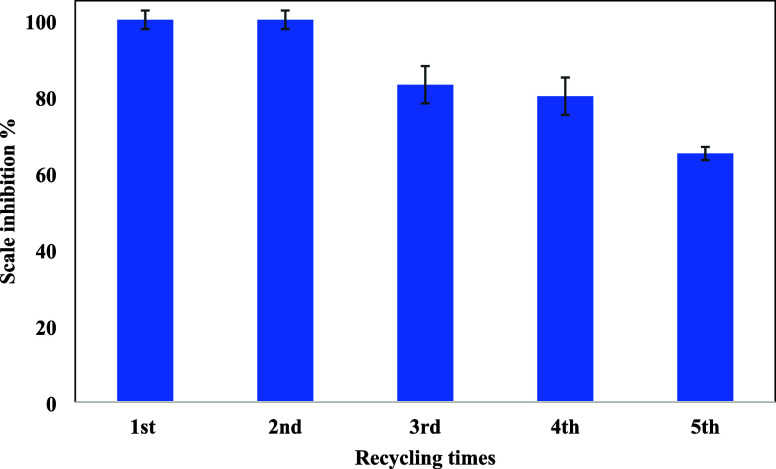
Recycling static inhibition test of SPION-TSC-PPEA at 100 ppm.

The recyclability of the composite was tested as a function of its efficiency in scale inhibition against the calcite scale with washing recycling times. The recyclability of the SPION composite was evaluated at 100 ppm concentration, as shown in Figure 6. After every run, a magnet was put beside the bottle to collect the nanoparticles. Then, the collected nanoparticles were washed with DI water and sonicated for 30 min to ensure they contained the least amount of sticked calcite scale traces if formed onto it. The efficiency was maintained until the fourth time of recycling at around 80% and then dropped to around 65% inhibition efficiency at the fifth time. The decrease in efficiency can be explained by the blockage of the functional groups of PPEA grafted onto the SPION surface by calcite scale nanocrystals.^7^ Also, it can be explained by the loss of PPEA from the SPION surface by the action of sonication of static inhibition test itself; however, this explanation has been falsified by ^31^P NMR analysis of the remaining solution of each, and no peaks were detected, as shown in Figure S1, in comparison to the original PPEA NMR chart in our latest publication.^20^ The PPEA residual absence in the solutions further confirmed the stability of the coating layer of PPEA onto SPION-TSC. This behavior correlates with our previous study on the inhibition of gypsum scaling.^23^

### 3.3 Calcium Compatibility Test

Calcium incompatibility can significantly undermine the effectiveness of scale inhibitors in oilfields, leading to a range of operational issues. Calcium ions can react with certain scale inhibitors, particularly those containing phosphonates or carboxylates, leading to the formation of inhibitor-calcium precipitates. These precipitates can themselves contribute to scaling, exacerbating the problem rather than mitigating it. The precipitation of calcium-inhibitor complexes can result in deposits that block pipelines, restrict flow, and damage equipment. These deposits can also form within the porous media of the reservoir, reducing permeability and potentially leading to formation damage. This can significantly decrease the efficiency of oil extraction processes.^4,29^

Calcium compatibility is one of the crucial factors in assessing SI in squeeze treatment applications, especially for phosphonated compounds compared to other chemical groups such as carboxylates or sulfonates.^4,31,39^ To determine the SI calcium compatibility, a series of tests were conducted at 80 °C in 30,000 ppm of NaCl and SI in concentrations of 100, 1000, 10,000, and 50,000 ppm, and a high Ca^2+^ concentration of 10000 ppm. The results were compared to the ATMP calcium compatibility results in Table 5. The SPION-TSC-PPEA SI different concentrations showed good calcium compatibility with adjacent different calcium ion concentrations, as shown in Table 6. However, with the highest concentration of both, some turbidity can be detected in the mixture with the progress of time under a set temperature. It is also noticeable that the turbidity can be also attributed to the high dispersity and high concentration of the SI itself, not due to calcium incompatibility alone, as shown in Table 7. Overall, SPION-TSC-PPEA SI showed greater calcium compatibility than that of commercially used ATMP.

**Table 5:** Calcium tolerance tests in 100, 1000, and 10,000 ppm of Ca^2+^ for ATMP

		appearance				
Ca^2+^ dose (ppm)	ATMP (ppm)	after mixing	30 min	1 h	4 h	24 h
100	100	clear	clear	clear	clear	clear
	1000	clear	clear	clear	clear	clear
	10,000	clear	clear	clear	clear	clear
	50,000	clear	clear	clear	clear	clear
1000	100	clear	clear	clear	precipitate	precipitate
	1000	clear	clear	clear	precipitate	precipitate
	10,000	clear	clear	clear	precipitate	precipitate
	50,000	clear	clear	precipitate	precipitate	precipitate
10,000	100	clear	hazy	hazy	precipitate	precipitate
	1000	clear	precipitate	precipitate	precipitate	precipitate
	10,000	clear	precipitate	precipitate	precipitate	precipitate
	50,000	clear	precipitate	precipitate	precipitate	precipitate

**Table 6:** Calcium tolerance tests in 100, 1000, and 10,000 ppm of Ca^2+^ for SPION-TSC-PPEA

		appearance				
Ca^2+^ dose (ppm)	SPION-TSC-PPEA (ppm)	after mixing	30 min	1 h	4 h	24 h
100	100	clear	clear	clear	clear	clear
	1000	clear	clear	clear	clear	clear
	10,000	clear	clear	clear	clear	clear
	50,000	clear	clear	clear	clear	clear
1000	100	clear	clear	clear	clear	clear
	1000	clear	clear	clear	clear	clear
	10,000	clear	clear	clear	clear	clear
	50,000	clear	clear	clear	clear	clear
10,000	100	clear	clear	clear	clear	clear
	1000	clear	clear	clear	clear	clear
	10,000	clear	clear	clear	clear	slightly hazy
	50,000	slightly hazy	slightly hazy	slightly hazy	slightly hazy	hazy

**Table 7:**
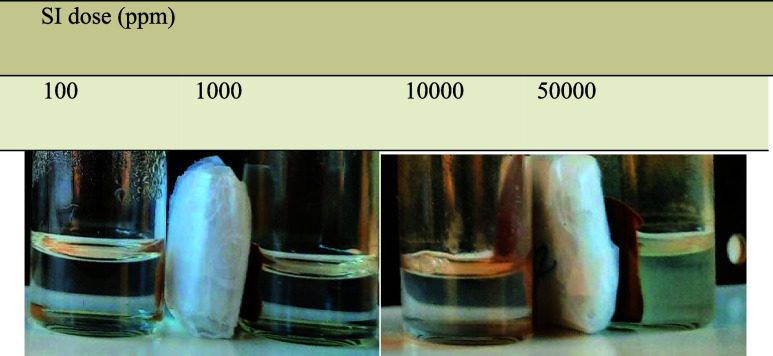
Visual inspection of calcium tolerance tests in 10,000 ppm of Ca^2+^ for SPION-TSC-PPEA after 24 h in 80 °C

### 3.4 High-Pressure Dynamic Tube-Blocking Test

**Figure 7:**
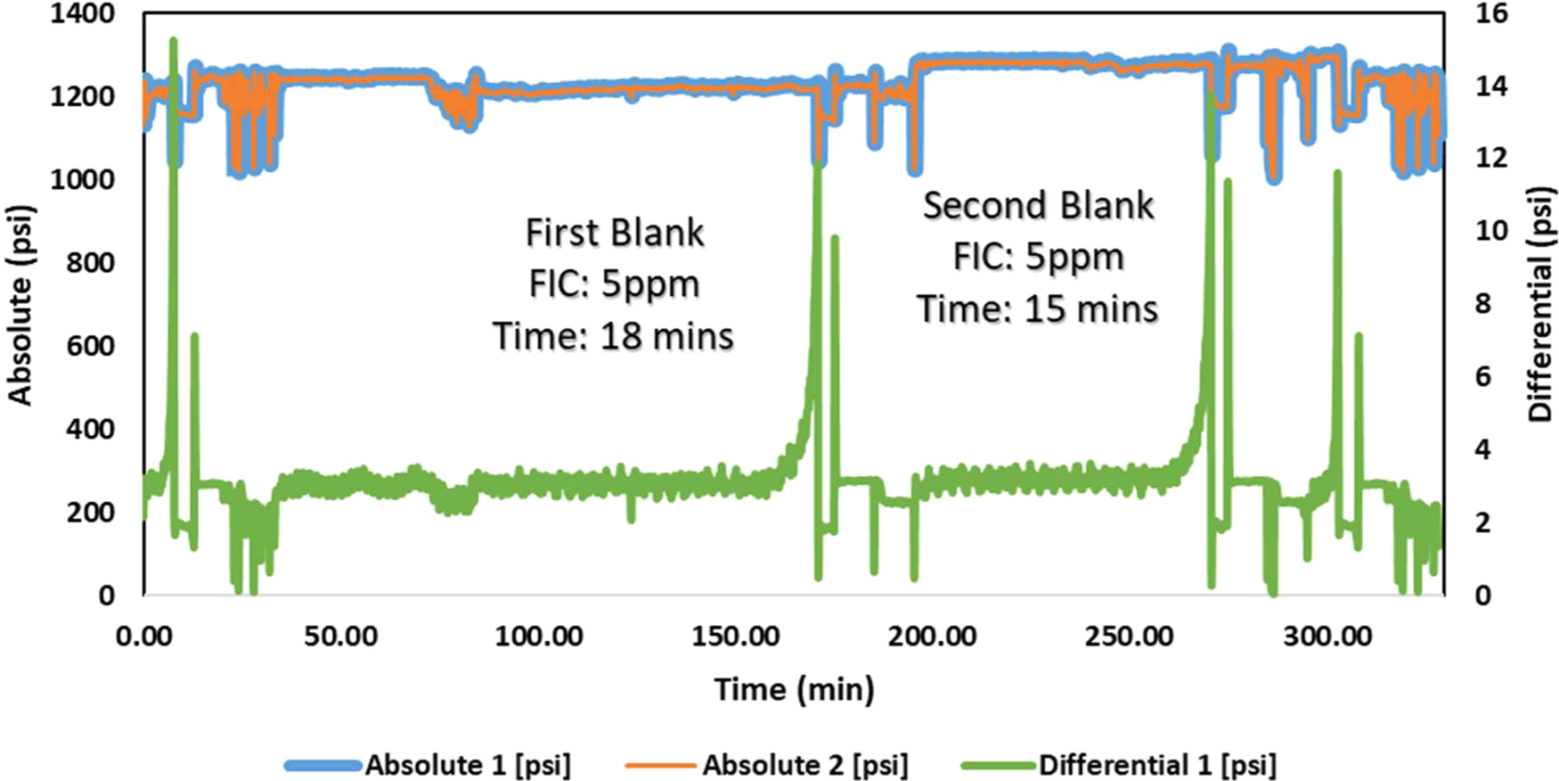
High-pressure dynamic tube-blocking test for PPEA against calcite.

**Figure 8:**
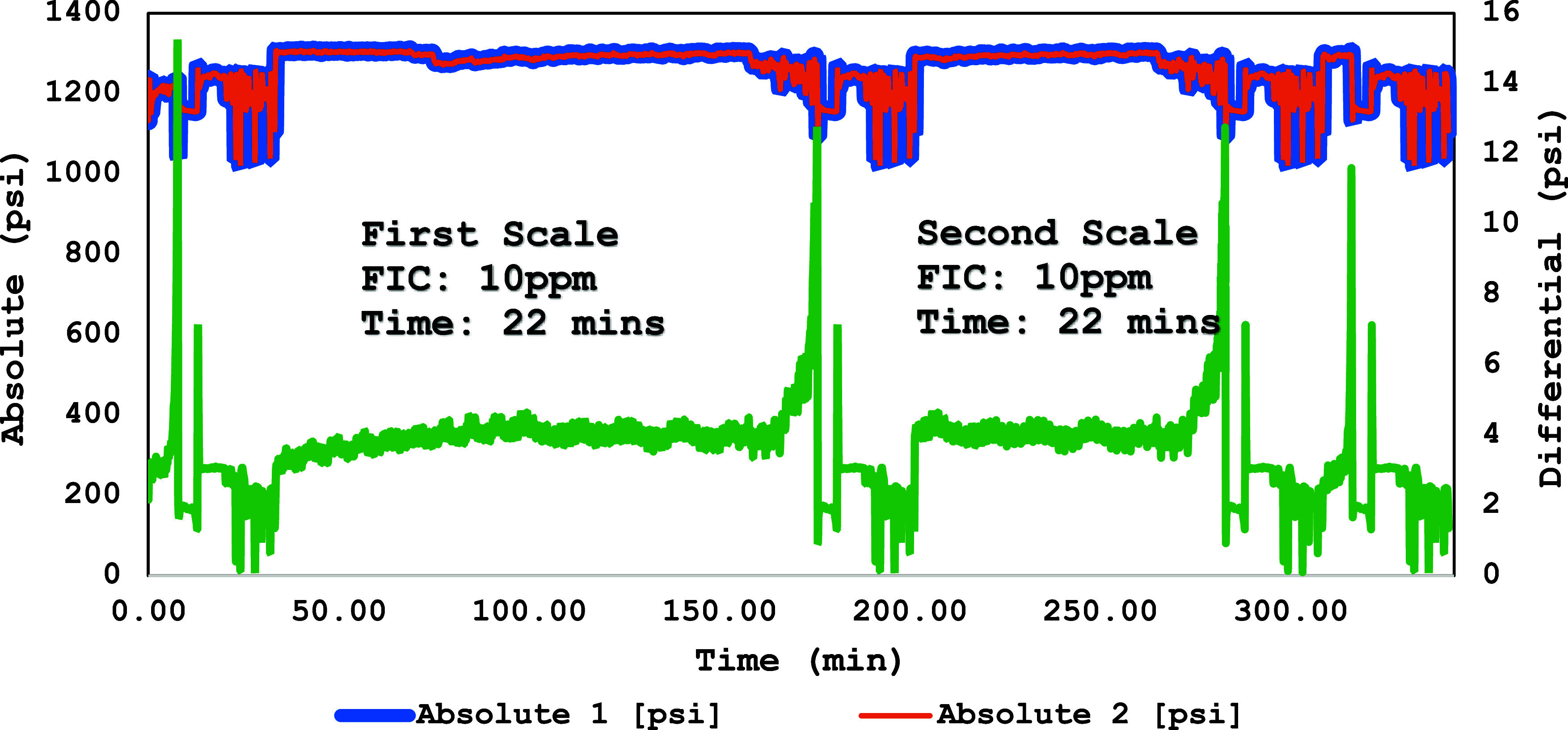
High-pressure dynamic tube-blocking test for SPION-TSC-PPEA against calcite.

**Figure 9:**
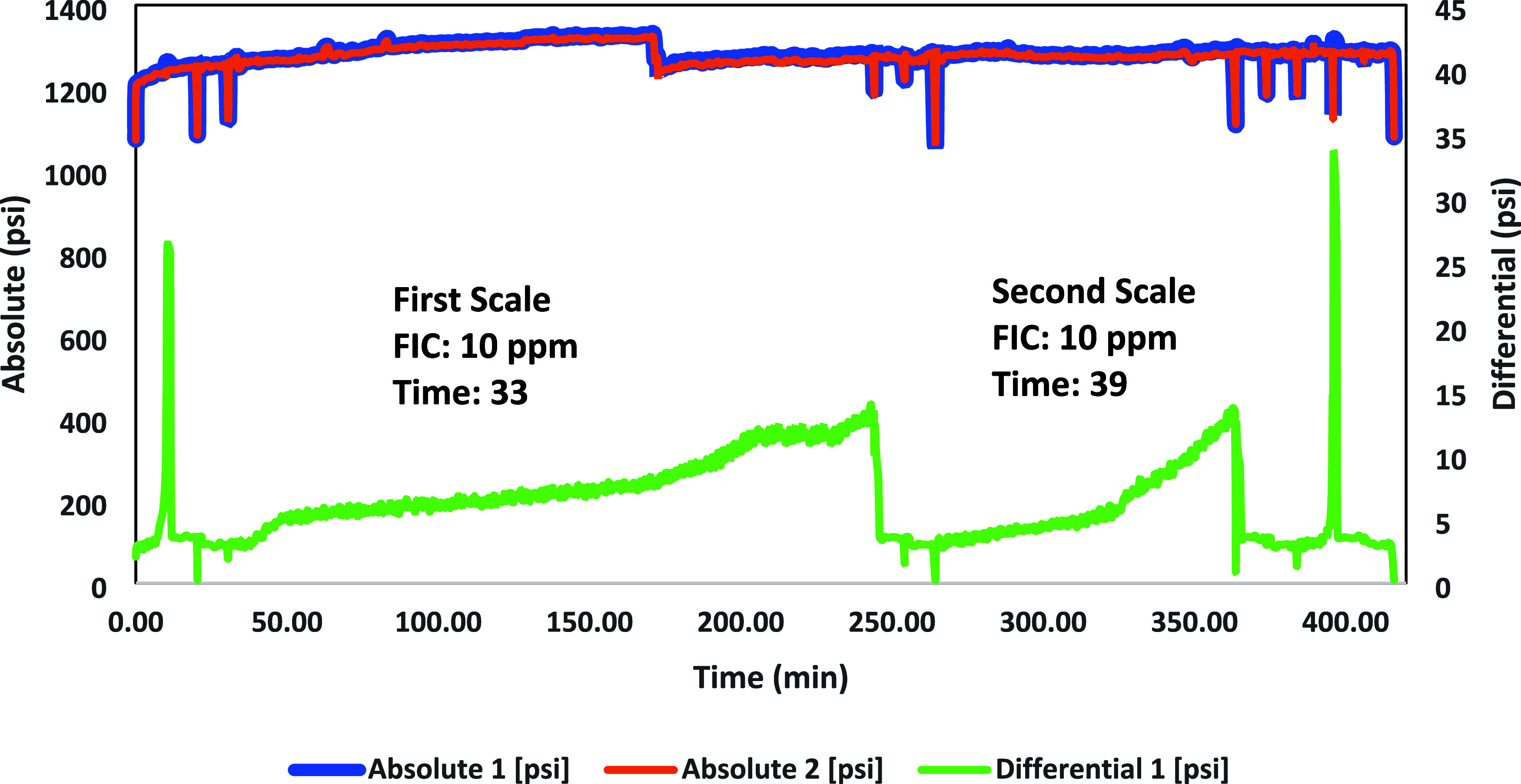
High-pressure dynamic tube-blocking test for ATMP against calcite.

**Figure 10:**
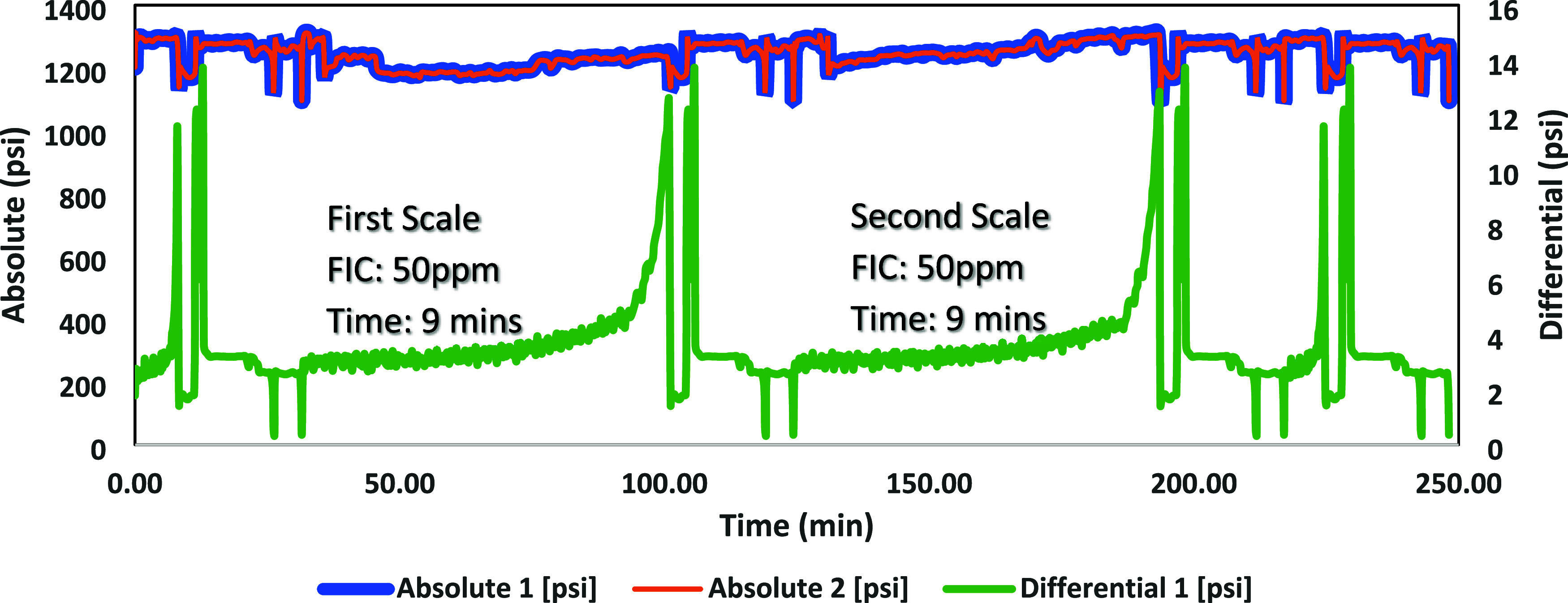
High-pressure dynamic tube-blocking test for PPEA against barite.

**Figure 11:**
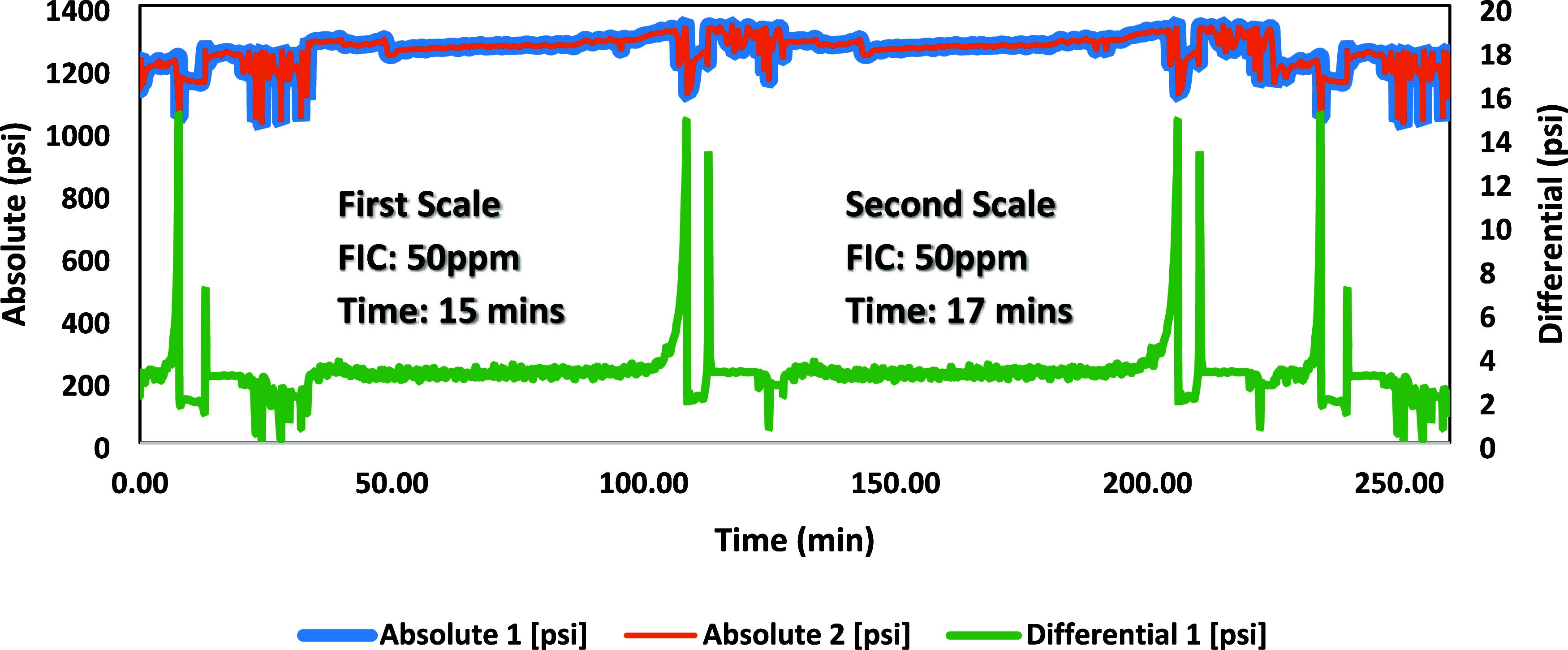
High-pressure dynamic tube-blocking test for SPION-TSC-PPEA against barite.

**Figure 12:**
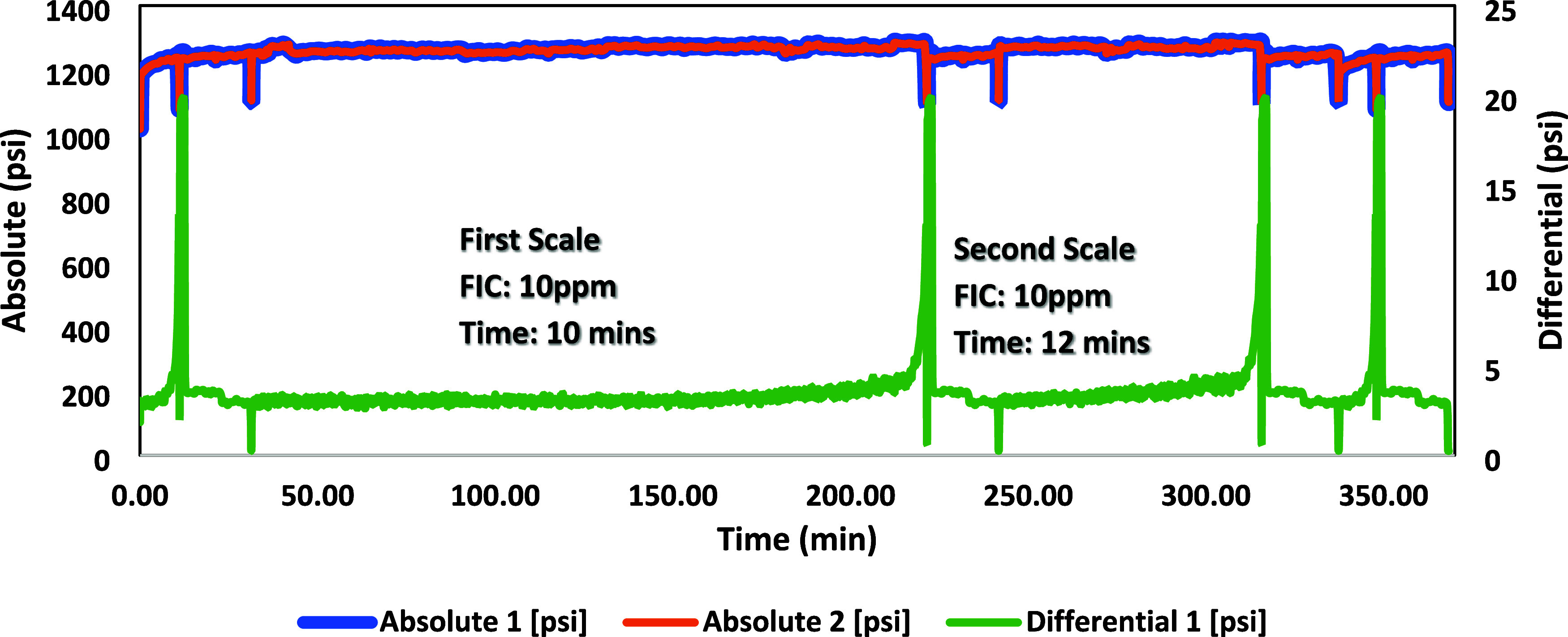
High-pressure dynamic tube-blocking test for ATMP against a barite scale.

A high-pressure dynamic tube-blocking rig test has been performed to investigate the ability of SPION composite to act as an SI at 100 °C and 80 bar in comparison with the main polymer (PPEA) and ATMP commercial SIs, as shown in Figures 7–12. The SPION-TSC-PPEA nanoparticles were dispersed in a 1000 ppm DI water solution with a pH of 4.5 to mimic a typical oil and gas reservoir pH.

Table 8 shows the results of the tested composite with the primary polymer and commercial ATMP SI against the calcite scale. The scale formation for the blank test of the calcite scale took 7–12 min, and it took 7–11 min for the barite scale. The primary SI polymer (PEPA) FIC was at 5 ppm. Upon coating onto SPION-TSC to produce SPION-TSC-PPEA, the efficiency decreased from FIC 5–10 ppm. The decrease can be illustrated by the reduction of the SI itself, as it is only represented on the surface of the nanoparticle composite, given that the polymer layer around the magnetic core compromises around 40 wt % of the overall nanocomposite.^23^ However, it is still acceptable, as it is the same FIC as the commercial SI (ATMP), and the recyclability would compensate for this efficiency reduction, as it can be used for further cycles. In addition, the composite has better calcium compatibility than ATMP. The overall results of the first and second scale formation times are correlated and accepted.

**Table 8:** Blank Tests and Fail Inhibitor Concentration (FIC) Values for Commercial and New Scale Inhibitors (SIs) for the Calcite Scale

SI	first blank	first scale test		second scale test		second blank
(1000 ppm)	time (min)	conc (ppm)	time (min)	conc (ppm)	time (min)	time (min)
ATMP	10	10	35	10	39	12
SPION-TSC-PPEA*	7	10	22	10	22	8
PPEA 50%	7	5	17	5	16	8

On the other hand, PPEA was proven less efficient against the barite scale as its FIC was 50 ppm, which can be illustrated by the reduction in the number of phosphonates in its repeating units. However, ATMP gave a better inhibition performance at 10 ppm, as shown in Table 9.

**Table 9:** Blank tests and fail inhibitor concentration (FIC) values for commercial and new scale inhibitors (SIS) for the barite scale

SI	first blank	first scale test		second scale test		second blank
(1000 ppm)	time (min)	conc (ppm)	time (min)	conc (ppm)	time (min)	time (min)
ATMP	11	10	10	10	12	10
PPEA 50%	8	50	9	50	9	8
SPION-TSC-PPEA	7	50	15	50	17	9

## 4 Conclusion

In conclusion, this study highlights the potential of (SPION-TSC-PPEA) as an effective and recyclable scale inhibitor for harsh environments in the oil and gas industry. The synthesized nanocomposite demonstrated high inhibition efficiency against calcite and moderate efficiency against barite in both static jar tests and high-pressure dynamic tube-blocking tests at challenging conditions of 80 bar and 100 °C. Importantly, the nanocomposite maintained its efficacy over five recycling cycles without losing its coating layer, showcasing its durability and reusability. Furthermore, it exhibited excellent calcium ion compatibility, addressing a common challenge associated with traditional scale inhibitors. The integration of SPIONs with phosphonated poly(ether amine) offers a promising green alternative that meets the stringent environmental regulations and operational demands of modern oilfield applications. This innovative approach not only improves scale management but also aligns with the industry’s move toward sustainable and environmentally friendly solutions. Future prospects of this study may include an ecotoxicology study to assess the environmental impact of the nanocomposite.

## Supporting Information Available

Figure S1: ^31^P NMR of recycled solution of SPION-TSC-PPEA. (PDF)
