# Supplementary material for: Phosphonated Polyetheramine-Coated Superparamagnetic Iron Oxide Nanoparticles: Study on the Harsh Scale Inhibition Performance of Calcium Carbonate and Barium Sulfate
Source: ACS Omega. 2024 Sep 29;9(40):42027–36. doi: 10.1021/acsomega.4c07018 (PMC11465520; doi:10.1021/acsomega.4c07018)
Supplement: Supplementary file 1 — ao4c07018_si_001.pdf [file ao4c07018_si_001.pdf]

Supporting Information

Phosphonated Polyetheramine-Coated Superparamagnetic Iron Oxide Nanoparticles: Study on Harsh Scale Inhibition Performance for Calcium Carbonate and Barium Sulfate

Ali H. Karaly[1]*, Malcolm A. Kelland[1], and Mohamed F. Mady[2]*

[1]*Department of Chemistry, Bioscience and Environmental Engineering, Faculty of Science and Technology, University of Stavanger, N-4036 Stavanger, Norway*

[2]*Department of Chemistry and Earth Sciences, College of Arts and Sciences, Qatar University, P.O. Box 2713, Doha, Qatar*

Corresponding Authors

E-mail addresses: ali.h.alkaraly@uis.no, mmady@qu.edu.qa

Table of Figures

Figure S1. ^{31}P NMR of recycled solution of SPIONs-TSC-PPEA, (D_2O, 162.00 MHz).	S-2

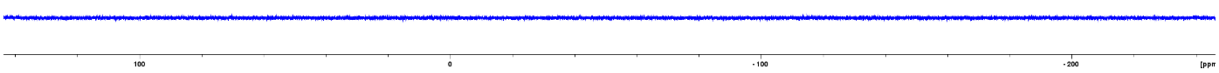

Figure S1. ^{31}P NMR of recycled solution of SPIONs-TSC-PPEA, (D_2O, 162.00 MHz).
